# Oxidative Stress Induces the Phosphorylation of NAD^+^ to NADP^+^ by NAD Kinase in Cultured Primary Rat Astrocytes

**DOI:** 10.1007/s11064-025-04588-4

**Published:** 2025-10-25

**Authors:** Johanna Elisabeth Willker, Patrick Watermann, Ralf Dringen

**Affiliations:** 1 Centre for Biomolecular Interactions Bremen, Faculty 2 (Biology/Chemistry), University of Bremen, P.O. Box 330440, 28334 Bremen, Germany; 2 Centre for Environmental Research and Sustainable Technologies, University of Bremen, Bremen, Germany

**Keywords:** Astrocytes, Glutathione, Nicotinamide coenzymes, NAD kinase, Oxidative stress

## Abstract

Astrocytes have important functions in the metabolism and antioxidative defence of the brain. Three redox pairs and the ratio of the reduced and oxidized partners in each pair are essential for astrocytic redox metabolism, GSx (glutathione (GSH) plus glutathione disulfide (GSSG)), NADx (NADH plus NAD^+^) and NADPx (NADPH plus NADP^+^). In order to elucidate the interactions between the three redox pairs in astrocytes, we first analysed the basal levels of the six redox co-substrates for cultured primary rat astrocytes by using sensitive and specific enzymatic cycling assays. In untreated cultures, the basal specific contents of GSx, NADPx and NADx were 44.7 ± 8.2 nmol/mg protein, 0.64 ± 0.09 nmol/mg protein and 2.91 ± 0.40 nmol/mg protein, with the reduced co-substrates accounting for 97 ± 3%, 37 ± 14% and 28 ± 10% of the total amounts, respectively. Exposure of cultured astrocytes to oxidative stress (100 µM H_2_O_2_ in the presence of the pentose-phosphate pathway inhibitor G6PDi-1) caused a rapid and severe but transient oxidation of GSH to GSSG. This increase was accompanied by a doubling of the total pool of NADPx on the expense of the cellular NADx pool, suggesting that NAD^+^ was phosphorylated to NADP^+^ under these conditions. Testing for NAD kinase (NADK) activity in lysates of cultured astrocytes revealed that the enzyme is present with a specific v_max_ activity of around 1 nmol/(min x mg protein) and has K_M_-values of 1.30 ± 0.19 mM for NAD^+^ and 2.71 ± 0.18 mM for ATP. Preincubation of astrocytes with thionicotinamide, the precursor for the cellular synthesis of the NADK inhibitor thio-NADP, prevented the transient oxidative stress-induced phosphorylation of NAD^+^ to NADP^+^. These data demonstrate that the NADPx pool can be increased in cultured astrocytes during oxidative stress by NADK-mediated phosphorylation of NAD^+^, providing experimental evidence for an additional interaction of the main astrocytic redox pairs during oxidative stress.

## Introduction

Astrocytes have many important functions in the brain as competent partners of neurons [1–3]. Especially their broad metabolic potential makes astrocytes an interesting cell type to study. For example, astrocytes have key functions in the metabolism of neurotransmitters [4], lactate [5], fatty acids [6], as well as in the defence against xenobiotics and oxidative stress [7]. Redox reactions are essential components of the main astrocytic metabolic pathways. Three main redox pairs are involved in astrocytic redox reactions by serving as reversible electron donors or electron acceptors, the nicotinamide adenine dinucleotide pair (NADx, consisting of oxidized NAD^+^ and reduced NADH), the nicotinamide adenine dinucleotide phosphate pair (NADPx, consisting of the oxidized NADP^+^ and the reduced NADPH) and the glutathione pair (GSx, consisting of reduced GSH and oxidized glutathione disulfide (GSSG)) [8].

The components of the NADx redox pair are well known for their participation in the cellular energy metabolism, but these molecules are also involved in deoxyribonucleic acid repair, as well as in the regulation of gene expression and blood flow, they contribute to calcium homeostasis, participate in intracellular signalling and play a role in cell survival and ageing [9–13]. During catabolism of energy substrates in astrocytes, electrons generated during oxidation of substrates are used to reduce NAD^+^ to NADH. Cytosolic NADH can then be oxidized back to NAD^+^ by the lactate dehydrogenase (LDH)-mediated reduction of pyruvate to lactate, while in mitochondria NADH is oxidized by the complex I of the respiratory chain. This is demonstrated by the strong basal lactate release from glucose-fed cultured astrocytes that is further accelerated in the presence of inhibitors of the respiratory chain [14, 15].

NADPH, the reduced molecule of the NADPx redox pair, serves mainly as electron donor for reductive biosyntheses and for antioxidative defence [16, 17]. In astrocytes, NADPH is predominately regenerated from NADP^+^ by the pentose phosphate pathway (PPP) [18–20], but several other cytosolic and mitochondrial enzymes can also provide electrons for NADP^+^ reduction, such as malic enzyme and NADP^+^-dependent isocitrate dehydrogenases [8, 21, 22].

The tripeptide GSH has many important functions in cells and serves as electron donor for the reduction of peroxides and radicals in addition to serving as a substrate for the detoxification of endogenous compounds and xenobiotics [7, 23, 24]. In astrocytes, GSH is present in millimolar concentrations [25]. In unstressed cultured astrocytes, cellular GSx represents almost exclusively GSH as transiently occurring GSSG is efficiently reduced back to GSH by the enzyme glutathione reductase which uses NADPH as electron donor [26]. However, GSSG accumulation is observed in astrocytes after induction of oxidative stress, for example by application of hydrogen peroxide [27], menadione [28, 29] or β-lapachone [20, 30]. Under such conditions the rapid glutathione peroxidase-mediated oxidation of GSH cannot be immediately compensated via NADPH-dependent reduction of GSSG by glutathione reductase [7].

The syntheses of GSH and NAD^+^ have been well studied for astrocytes. GSH is synthesized from the amino acid substrates glutamate, cysteine and glycine by the consecutive reactions catalyzed by γ-glutamylcysteine ligase and GSH synthetase [7], while NAD^+^ is synthesized from nicotinamide, phosphoribosyl pyrophosphate and adenosine triphosphate (ATP) [11, 31]. In contrast, hardly any information is available on the formation of NADP^+^ in astrocytes. It can be assumed that NAD kinase (NADK) produces NADP^+^ by phosphorylation of NAD^+^ in astrocytes, as shown for many other mammalian cell types [32, 33]. For the brain, the presence of NADK has been shown and some kinetic properties of this enzyme have been reported [34, 35].

Although substantial amounts of evidence have been provided on the modulation of the individual redox pairs GSx, NADx and NADPx by given stressors, metabolic inhibitors or starvation conditions, little information is available on the direct interplay between the three main redox pairs in astrocytes. Our new study aims to gain additional knowledge on the time lines of alterations of all three redox pairs and their ratios under transient oxidative stress conditions. By using sensitive enzymatic cycling assays, we first determined the basal levels of GSx, NADx and NADPx as well as the percental contribution of the respective reduced and oxidized components of these redox pairs. Then, we tested for the consequences of oxidative stress on the three redox pairs in cultured astrocytes. Oxidative stress caused a reversible oxidation of GSx and NADPx in astrocytes and led to a transient increase of the NADPx pool on the expense of the NADx pool which was inhibited by preincubation of the cells with thionicotinamide to inactivate NADK. These data demonstrate that the available cellular NADPx content can rapidly be doubled by NADK-mediated phosphorylation of NAD^+^. These findings provide additional evidence for the strong interplay between the three main redox pairs in astrocytes during oxidative stress.

## Methods

### Materials

Fetal calf serum (FCS), glucose-6-phosphate, magnesium chloride, 3-(4,5-dimethyl 2-thiazolyl)−2,5-diphenyl-2H-tetrazolium bromide (MTT), the redox cyclers phenazine ethosulfate (PES) and phenazine methosulfate (PMS), 5,5’-dithiobis (2-nitrobenzoic acid) (DTNB), GSSG, glutathione reductase and tris(hydroxymethyl)aminomethane (Tris) were purchased from Sigma-Aldrich (Steinheim, Germany). Dulbecco´s modified Eagle´s medium (DMEM; containing 25 mM glucose), penicillin G/streptomycin sulfate solution and thionicotinamide were obtained from Thermo Fisher Scientific (Schwerte, Germany). Dimethyl sulfoxide (DMSO), sulfosalicylic acid, NAD^+^, NADH and NADPH were bought from AppliChem (Darmstadt, Germany). ATP, lactate dehydrogenase (LDH) and yeast glucose-6-phosphate dehydrogenase (G6PDH) were purchased from Roche Diagnostics (Mannheim, Germany). Nicotinamide and 2-vinylpyridine were from Fluka (Steinheim, Germany). Hydrogen peroxide (H_2_O_2_) was obtained from Merck (Darmstadt, Germany). G6PDi-1 was from Cayman Chemical (Tallin, Estonia). All other basal chemicals were purchased from Sigma-Aldrich (Steinheim, Germany), Roth (Karlsruhe, Germany) or Merck (Darmstadt, Germany). Sterile cell culture consumables and unsterile 96-well plates were obtained from Sarstedt (Nümbrecht, Germany).

### Primary Astrocyte-Rich Cultures

The cell experiments of this study were performed on astrocyte-rich primary cultures that had been prepared from the brains of newborn Wistar rats as described previously [36]. The rats were obtained from Charles River Germany (Sulzfeld, Germany) and were treated in accordance with the animal welfare acts of the State of Bremen, Germany and Europe. The harvested cells were plated in 1 mL culture medium (90% DMEM containing 25 mM glucose, 44.6 mM sodium bicarbonate, 1 mM pyruvate, 20 U/mL penicillin G, 20 µg/mL streptomycin sulfate, supplemented with 10% FCS) in a density of 300,000 cells per well in wells of 24-well plates. The primary astrocytes were cultured in a humidified atmosphere at 37 °C with 10% CO_2_ in a Sanyo (Osaka, Japan) CO_2_ cell incubator. The medium was replaced every 7 days and on the day before the experiment. The cultures reach confluency after around 12 days in culture. Confluent astrocyte cultures aged 14–28 days were used for the experiments performed for this study. These cultures contain mainly glial fibrillary acidic protein-positive astrocytes and only low numbers of microglial cells and oligodendrocytes [37].

### Experimental Incubations

Astrocyte cultures were washed twice with 1 mL amino acid- and energy substrate-free incubation buffer (IB; 20 mM HEPES, 145 mM NaCl, 1.8 mM CaCl_2_, 1 mM MgCl_2_, 5.4 mM KCl, 0.8 mM Na_2_HPO_4_, pH adjusted to 7.4 at 37 °C with NaOH) and incubated with 200 µL incubation medium (IB with 5 mM D-glucose that was supplemented with substrates and/or modulators of metabolic processes as indicated in the figure legends) for given incubation periods at 37 °C in a humidified atmosphere of an incubator. Incubation of cultured astrocytes with 5 mM glucose in IB enables the cells to consume glucose with maximal glycolytic productions of lactate and pyruvate for several hours [14]. After the given incubation period, the incubation medium was harvested to determine the extracellular LDH activity as indicator for a potential loss in cell viability and the remaining extracellular concentration of H_2_O_2_. The cells were washed twice with 1 mL cold (4 °C) phosphate-buffered saline (PBS; 10 mM potassium phosphate buffer pH 7.4 containing 150 mM NaCl) and lysed for the quantification of the cellular contents of NADx, NADPx or GSx.

### Determination of the Cellular NADx and NADPx Contents

The washed cells were lysed in 500 µL extraction buffer (20 mM NaHCO_3_, 100 mM Na_2_CO_3_, 15 mM nicotinamide, 0.05% (w/v) Triton X-100) for 10 min on ice in the dark. Cell lysates of two identically treated wells were pooled and ultrasonicated. The oxidized nicotinamide cofactors were destroyed in separate aliquot parts of the lysate by a 30 min incubation at 60 °C in the dark [38]. The contents of NAD^+^, NADH and NADx (sum of NAD^+^ plus NADH) were determined by a cycling assay measuring the reduction of MTT in the presence of 3 mM PMS, 167 mM lactate and 45 U/mL LDH, while the contents of NADP^+^, NADPH and NADPx (sum of NADP^+^ plus NADPH) were determined from the same lysate by a cycling assay measuring the reduction of MTT in the presence of 3 mM PES, 1 mM glucose-6-phosphate and 30 U/mL G6PDH, as described recently [38]. The MTT formazan formation was monitored for 10 min by the increase in absorbance at 570 nm with the Multiskan Sky microplate spectrophotometer (Thermo Fisher Scientific, Bremen, Germany). The contents of NADx, NADH, NADPx and NADPH per sample were calculated from the linear calibration curves obtained for NADH (0–100 pmol/well) or NADPH standards (0–60 pmol/well) correspondingly [38]. The specific cellular NADx or NADPx contents were calculated by normalising the content of the cellular nicotinamide cofactors per well to the respective initial cellular protein content. The sum of the specific values for NADx and NADPx was calculated and is presented as NAD(P)x. Accordingly, NAD(P)^+^ represents the sum of NAD^+^ plus NADP^+^, while NAD(P)H refers to the sum of NADH plus NADPH.

### Determination of the Cellular GSx and GSSG Contents

The cellular GSx (GSH plus 2x GSSG) and GSSG contents were quantified based on the method by Tietze [39] adapted for microtiter plates as described before [40]. Cell lysis was performed with 200 µL 1% (w/v) sulfosalicylic acid for 10 min on ice. To measure the GSx content, 10 µL of the lysates or of a GSSG standard ranging from (0–400 pmol GSx/10 µL) were transferred to wells of a 96-well plate and diluted with 90 µL of water. The reaction was started by the addition of 100 µL of a reaction mixture (0.1 M sodium phosphate buffer, 1 mM ethylenediamide tetra acetic acid (EDTA), 0.4 mM NADPH, 0.3 mM DTNB, 0.05 U glutathione reductase, pH 7.5). The formation of thionitrobenzoate was monitored for 10 min by the increase in absorbance at 405 nm with the Multiskan Sky microplate spectrophotometer. To determine the GSSG content of lysates, GSH in the samples was first removed by derivatization with 2-vinylpyridine [36]. For this, 130 µL of the lysate sample or of GSSG standard (ranging from 0 to 400 pmol GSx/10 µL) were mixed with 5 µL 97% 2-vinylpyridine and the pH was adjusted to pH 6 by the addition of 0.2 M Tris base solution. After thorough mixing, the samples were incubated at room temperature for 60 min. Remaining GSSG in the 2-vinylpyridine-treated samples was then quantified as GSx as described above. The contents of GSx and GSSG in the samples were determined by comparison to the linear calibration curve obtained for GSSG standards (0–400 pmol GSx/well). The specific cellular GSx and GSSG contents were calculated by normalising the contents determined to the initial cellular protein content of the respective culture.

### Determination of the NAD Kinase Activity

To determine the activity of the NADK, cultured astrocytes in wells of a 24-well plate were washed two times with 1 mL ice cold PBS and once with 1 mL ice cold hypotonic lysis buffer (20 mM Tris-HCl, pH 7.5) before the cells in one well were lysed with 200 µL lysis buffer for 10 min on ice. The cells were scraped off and the lysate was centrifuged at 12,100 *g* for 5 min. If not stated otherwise, 200 µL of the resulting supernatant were mixed with 800 µL reaction mixture to generated final concentrations of 110 mM Tris-HCl pH 7.8, 10 mM MgCl_2_, 2 mM NAD^+^ and 6 mM ATP. After 30 min of incubation at 37 °C, if not stated differently, 100 µL of the reaction solution were harvested and mixed with 100 µL extraction buffer (20 mM NaHCO_3_, 100 mM Na_2_CO_3_, 15 mM nicotinamide, 0.05% (w/v) Triton X-100) to stop the reaction. The NADP^+^ content that had been generated during the incubation was then determined by the NADPx assay described above, with a modified range of NADPH standards (0–100 pmol NADPH/well) that contained the appropriate amounts of lysis and extraction buffers. The specific activity of the NADK was calculated by normalizing the NADPx formation to the protein content of the lysate supernatant of the respective culture.

### Determination of H_2_O_2_ Detoxification

The clearance of extracellular H_2_O_2_ by cultured astrocytes was measured by a slight modification of the peroxide assay previously described [25, 41] which is based on the peroxide-mediated oxidation of ferrous iron. From the incubation media that initially contained 100 µM H_2_O_2_, 10 µL samples were collected after the given incubation periods and diluted with 170 µL 25 mM H_2_SO_4_ in wells of a microtiter plate before 180 µL of peroxide reaction mixture (222 mM sorbitol, 28 mM H_2_SO_4_, 556 mM (NH_4_)_2_Fe(SO_4_)_2_, 222 µM xylenol orange) were added to each well. After an incubation at room temperature for 45 min the absorbance of the ferric xylenol orange complex at 540 nm was measured as indicator for the remaining concentration of peroxide [25]. The half-time for the peroxide was calculated from the cell-dependent first order clearance of the peroxide and was normalized on the cellular protein content by calculating the specific detoxification rate constant D as previously described [41].

### Cell Viability and Protein Determination

The extracellular activity of the cytosolic enzyme LDH was determined as an indicator of potential toxicity of a treatment, as described in detail before [36]. The oxidation of NADH in the presence of pyruvate was monitored at 340 nm and compared with the total initial cellular LDH activity after Triton X-100 lysis. The initial cellular protein content per well of untreated cells was determined by the Lowry method [42], using bovine serum albumin as standard protein. The protein standards used for quantifying the protein content of the lysate supernatant used to measure the NADK activity contained the appropriate amounts of lysis buffer.

### Data Analysis and Statistical Analysis

Quantitative data are presented as means ± standard deviation (SD). If not stated otherwise, the experiments were performed on three independently prepared astrocyte cultures. Normal distribution was confirmed for data sets derived from more than 5 independent experiments by the Kolomogorov-Smirnov test, while normal distribution was assumed for all data sets derived from less independent experiments. Statistical analysis of multiple groups of data was performed as ANOVA (followed by Bonferroni post-hoc test) and the level of significance of differences is indicated by *p < 0.05, **p < 0.01 and ***p < 0.001. For the comparison of two datasets a paired one-tailed t-test was done. Significances of differences are given by ^#^*p* < 0.05, ^##^*p* < 0.01 and ^###^*p* < 0.001, as indicated in the figure legends. *p* ≥ 0.05 was considered as not significant and is not indicated in the figures. For all statistical calculations the software GraphPad InStat 3 (GraphPad, Boston, USA) was used. Regression curves, analysis of significance of the coefficients for the age dependency data as well as Michaelis-Menten curves for the NADK characterisation were calculated using SigmaPlot 14.5 (Systat, Inpixon, Düsseldorf, Germany).

## Results

### Basal Contents of Redox Co-substrates in Cultured Astrocytes

In order to investigate potential alterations in the three main redox pairs in astrocytes, we first quantified the basal contents of the redox co-substrates in untreated astrocyte cultures by using sensitive enzymatic cycling assays [36, 38]. The average specific contents of GSx, NADPx and NADx from a large number of individual experiments was determined as 44.69 ± 8.21 nmol/mg protein, 0.64 ± 0.09 nmol/mg protein and 2.91 ± 0.40 nmol/mg protein, with the reduced partners accounting for 97 ± 3%, 37 ± 14% and 28 ± 10% of the total amounts, respectively (Table 1). Compared to the average specific cellular NADx content (Table 1), the average content of the GSx pool is around 15-times higher, while the average NADPx pool represents only around 20% of the NADx pool.

**Fig. 1:**
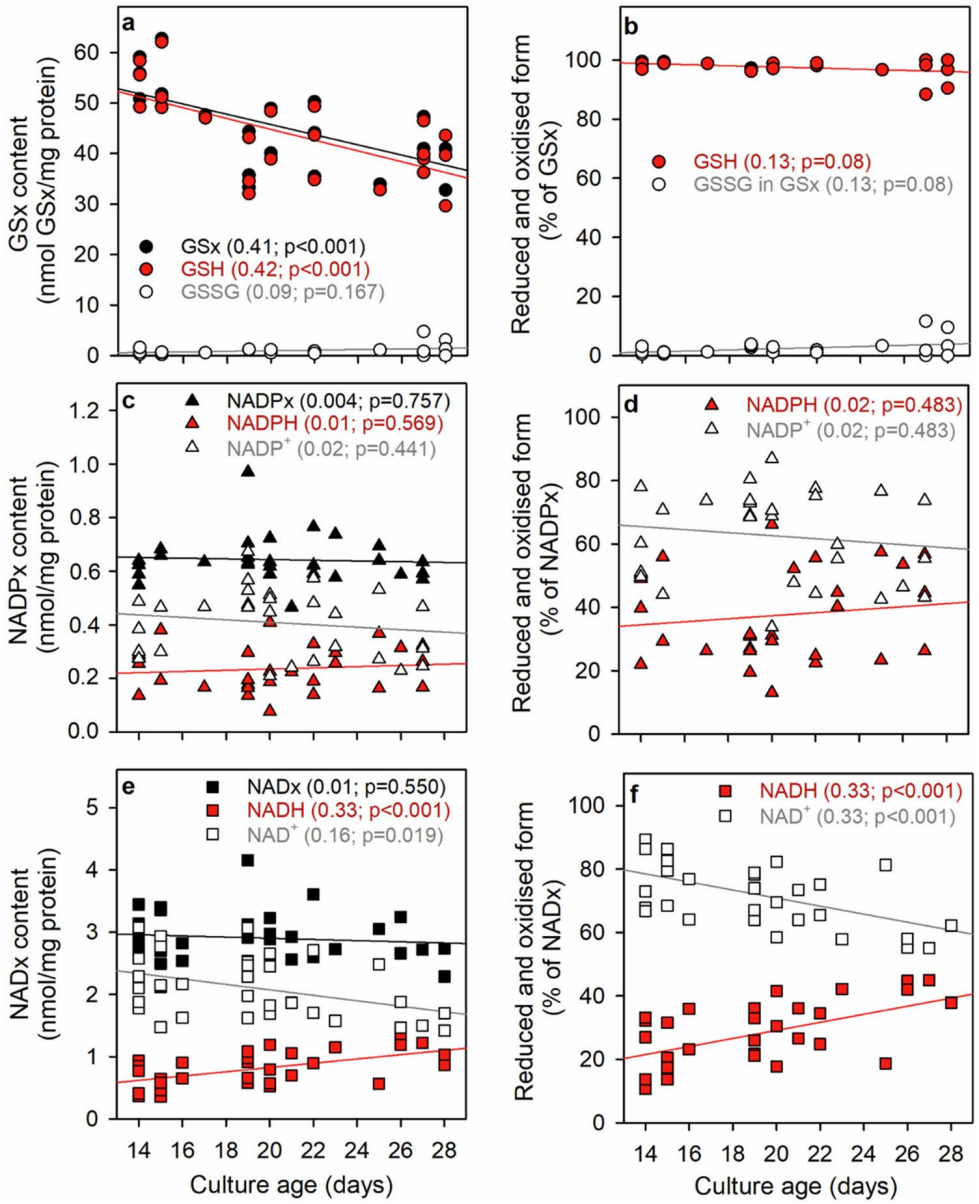
Culture age-dependency of the cellular contents of GSx (**a**, **b**), NADPx (**c**, **d**) and NADx (**e**, **f**) of untreated astrocyte cultures. Shown are the specific amounts of total, reduced and oxidized co-substrates (**a**,** c**,** e**) as well as the percental amounts of the reduced and oxidized species (**b**,** d**,** f**). Each panel contains lines showing the linear regression analysis in the colours of the respective symbols and gives the correlation parameters r² and the p-values of the predicted slopes in the colours of the respective lines. The total number of individual experiments and the number of independently prepared cultures that were used for the measurements as well as the average protein contents per well are given in Table 1

To test for a potential age dependency of the GSx, NADPx and NADx contents of astrocyte cultures, the specific contents of the three redox pairs and of their reduced and oxidized components (Fig. 1a, c, e) as well as their respective percental contribution (Fig. 1b, d, f) were correlated with the age of the respective culture. For all substances investigated, some variations in their specific contents between the data obtained for individual measurements were observed (Fig. 1), consistent with the low correlation parameters (given in the panels of Fig. 1) and the high SD values calculated for the average values (Table 1). Regression analysis of the data revealed that among the investigated parameters the specific GSx content (Fig. 1a) declined significantly by around 30% with an increasing culture age between 14 and 28 d (Fig. 1a), while the specific NADPx and NADx values were not affected by the culture age (Fig. 1c, e). Also, the specific contents of NADPx, NADP^+^ and NADPH were not obviously altered with the increasing culture age (Fig. 1c, d), while a strong age-dependency was found for the individual components of the NADx redox pair with an age-dependent decline in NAD^+^ contents and an age-dependent increase in NADH contents (Fig. 1e, f).

**Table 1:** Specific cellular contents of the three main redox pairs in untreated astrocyte primary cultures

Compound	Content per well (pmol/well)	Specific content (nmol/mg protein)	Content (%)	Number of experiments (independent cultures)
GSx	6561 ± 885	44.69 ± 8.21	100	23 (20)
GSH	6406 ± 961	43.65 ± 8.60	97 ± 3	23 (20)
GSSG (as GSx)	155 ± 155	1.03 ± 1.03	3 ± 3	23 (20)
NADPx	95 ± 21	0.64 ± 0.09	100	28 (19)
NADPH	35 ± 16	0.24 ± 0.08	37 ± 14	28 (19)
NADP^+^	60 ± 20	0.41 ± 0.13	63 ± 14	28 (19)
NADx	389 ± 105	2.91 ± 0.40	100	34 (24)
NADH	109 ± 49	0.80 ± 0.27	28 ± 10	34 (24)
NAD^+^	280 ± 87	2.11 ± 0.49	72 ± 10	34 (24)

The data represent the means ± SD of values from a total of n independent experiments that had been performed on the given number of different independently prepared cultures (given in brackets). The results of the individual measurements are shown in Fig. 1. Initial protein contents amounted to 150 ± 27 µg/well (*n* = 23), 150 ± 29 µg/well (*n* = 28) and 133 ± 28 µg/well (*n* = 34)

### Consequences of a G6PDi-1 Application on the Hydrogen Peroxide Clearance by Astrocytes

**Fig. 2:**
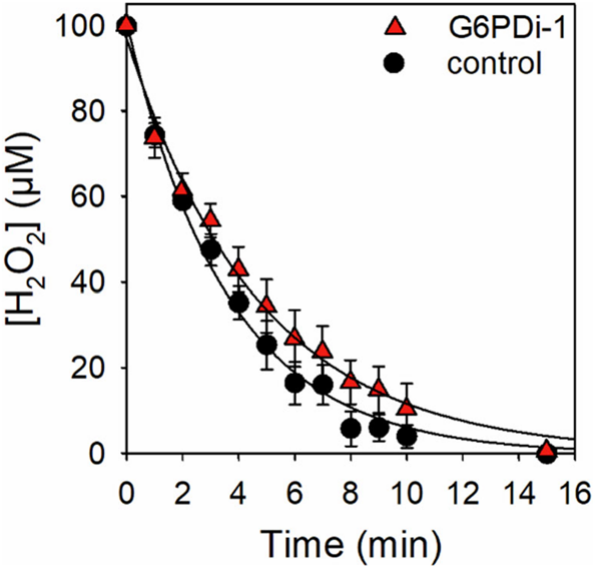
Clearance of exogenous H_2_O_2_ by cultured astrocytes in the absence or the presence of the glucose-6-phosphate dehydrogenase inhibitor G6PDi-1. The cells were incubated with 100 µM H_2_O_2_ without or with 30 µM G6PDi-1 for up to 15 min. The initial protein content of the cultures was 118 ± 13 µg/well. The data presented are means ± SD of values from experiments performed on three independently prepared astrocyte cultures

In order to investigate the effects of oxidative stress on the cellular contents of the three main redox pairs in astrocytes, the cultures were exposed to H_2_O_2_ in the absence or the presence of the specific G6PDH inhibitor G6PDi-1 [19, 43]. In the absence of the inhibitor the exogenous peroxide disappeared rapidly (Fig. 2) with a half-time of 2.51 ± 0.30 min and a specific peroxide detoxification rate constant (D) of 3.42 ± 0.16 (min x mg protein)^−1^ (Table 2). This rapid clearance of the exogenous peroxide was slowed in the presence of G6PDi-1 (Fig. 2) as demonstrated by the significant increase in the half-time by 30% and the significant decrease in the D-value by 25% (Table 2).

**Table 2:** Half-time and specific peroxide detoxification rate constants (D) for the clearance of exogenous H_2_O_2_ by astrocytes in the absence or the presence of G6PDi-1

		Control	G6PDi-1
Half-time of H_2_O_2_	min	2.51 ± 0.30	3.27 ± 0.53^#^
	% of control	100 ± 12	130 ± 21^#^
D-value	(min x mg protein)^−1^	3.42 ± 0.16	2.64 ± 0.17^##^
	% of control	100 ± 5	77 ± 5^##^

The values were calculated from the data presented in Fig. 2. The significance of differences (t-test) between the two incubation conditions is indicated as ^#^*p* < 0.05 and ^##^*p* < 0.01

### Consequences of Oxidative Stress on the Main Redox Pairs in Astrocytes

**Fig. 3:**
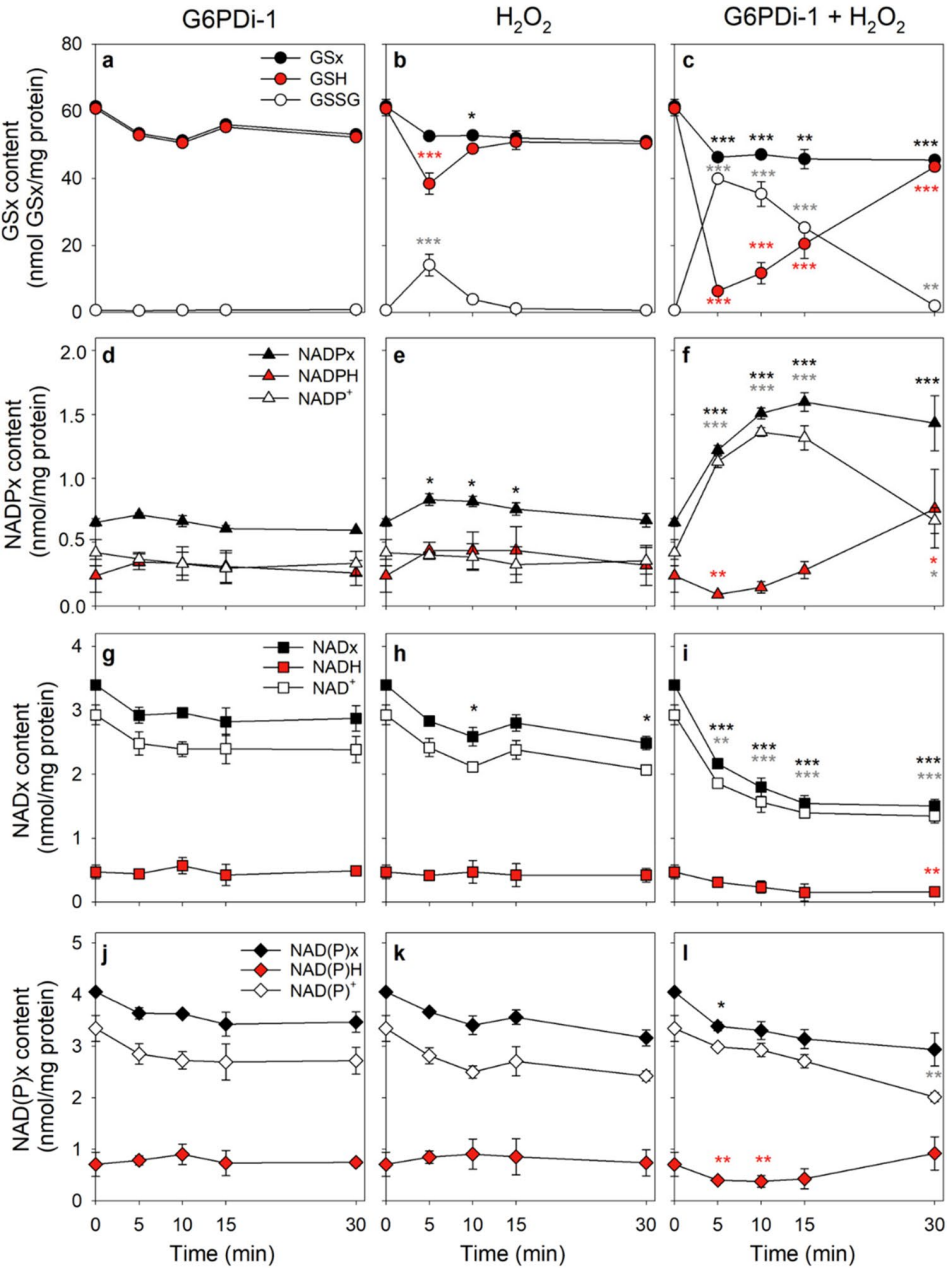
Effects of H_2_O_2_-induced oxidative stress on the main redox pairs of cultured astrocytes. The cells were incubated with 100 µM H_2_O_2_ and/or 30 µM G6PDi-1 for up to 30 min. The cellular contents of GSx (**a-c**), NADPx (**d-f**), and NADx (**g-i**) as well as the contents of the reduced and oxidized partners of each redox pair were measured. In addition, the contents of NAD(P)x (sum of NADPx plus NADx), NAD(P)H (sum of NADPH plus NADH) and NAD(P)^+^ (sum of NADP^+^ plus NAD^+^) were calculated (**j-l**). The absence of any significant increase in extracellular LDH activity after 30 min of incubation (data not shown) revealed that the cell viability was not compromised by the treatments. The initial specific GSx content was 61.5 ± 2.1 nmol/mg protein (with GSH accounting for 99% of the GSx pool), the initial specific NADPx content was 0.66 ± 0.03 nmol/mg protein (with NADPH accounting for 36% of the NADPx pool), and the initial specific NADx content was 3.40 ± 0.05 nmol/mg protein (with NADH accounting for 14% of the NADx pool). The initial protein content of the cultures was 108 ± 7 µg per well. The data presented are means ± SD of values from experiments performed on three independently prepared cultures. The significance of differences (ANOVA) compared to the data obtained for the initial timepoint (0 min) is indicated by *p < 0.05, **p < 0.01 and ***p < 0.001 in the colour of the symbols used to indicate the specific content of the quantified substance

During incubation of cultured astrocytes for up to 30 min with G6PDi-1 (Fig. 3a, d, g, j) in the absence of peroxide, the specific cellular contents of GSx, NADPx, NADx and NAD(P)x as well as the contribution of the two partners of each redox pair were not altered significantly compared to the respective initial values. In contrast, the application of 100 µM H_2_O_2_, caused a significant and transient decrease in the cellular GSH content during the first 5 min of incubation (from 61 to 38 nmol/mg protein) and a corresponding increase in the cellular GSSG content (from 1 to 14 nmol/mg protein) (Fig. 3b), that was accompanied with a small increase (by 27%) in the cellular NADPx content (Fig. 3e). Meanwhile the specific cellular NADx content (Fig. 3h) and the sum of NADx plus NADPx (NAD(P)x) was rather unaffected (Fig. 3k). Also, the contents of the reduced and oxidized partners of the redox pairs NADPx (Fig. 3e) and NADx (Fig. 3h) remained unaltered.

Severe differences to those results were observed, when cultured astrocytes had been exposed to 100 µM H_2_O_2_ in the presence of G6PDi-1 (Fig. 3c, f, i, l). Under these conditions, the initial high content of cellular GSH was found almost completely oxidized to GSSG within 5 min (Fig. 3c), while the cellular NADPx content was strongly increased during the first 10 min of incubation (from 0.7 to 1.5 nmol/mg protein) (Fig. 3f) on the expense of the cellular NADx content (Fig. 3i). The NAD(P)x content was hardly affected (Fig. 3l). For the initial 10 min of incubation, the oxidized partners of the three redox pairs accounted almost completely for the total contents of GSx (Fig. 3c), NADPx (Fig. 3f), NADx (Fig. 3i) as well as of NAD(P)x (Fig. 3l). After 15 min of incubation, the cellular NADPx content had reached a maximal value of 1.6 nmol/mg protein that represented around 250% of the initial content (Fig. 3f). A matching decline in cellular NADx content to 45% of the initial content (Fig. 3i) was found, resulting in almost identical specific contents of NADPx and NADx in the cells. The calculated NAD(P)x contents decreased slightly during the initial 5 min of incubation with peroxide plus G6PDi-1, while around 20% of the initial NAD(P)x content were lost during the entire 30 min incubation (Fig. 3l).

During incubation of astrocytes with H_2_O_2_ and G6PDi-1 for more than 5 min, the accumulated GSSG was reduced to GSH again and the initial high ratio of GSH to GSSG was fully reestablished within 30 min after application of the peroxide (Fig. 3c). Similarly, after 10 min of incubation, the specific NADPH content increased slowly and represented with 0.76 nmol/mg protein half of the total NADPx content and thrice the initial content (0.24 nmol/mg protein) of NADPH after 30 min of incubation (Fig. 3f). In contrast, the cellular NADH content remained lowered throughout the entire incubation at around 1.5 nmol/mg protein (Fig. 3i), while a slight recovery of the NAD(P)H value was observed (Fig. 3l), due to the increase in cellular NADPH content (Fig. 3f).

**Fig. 4:**
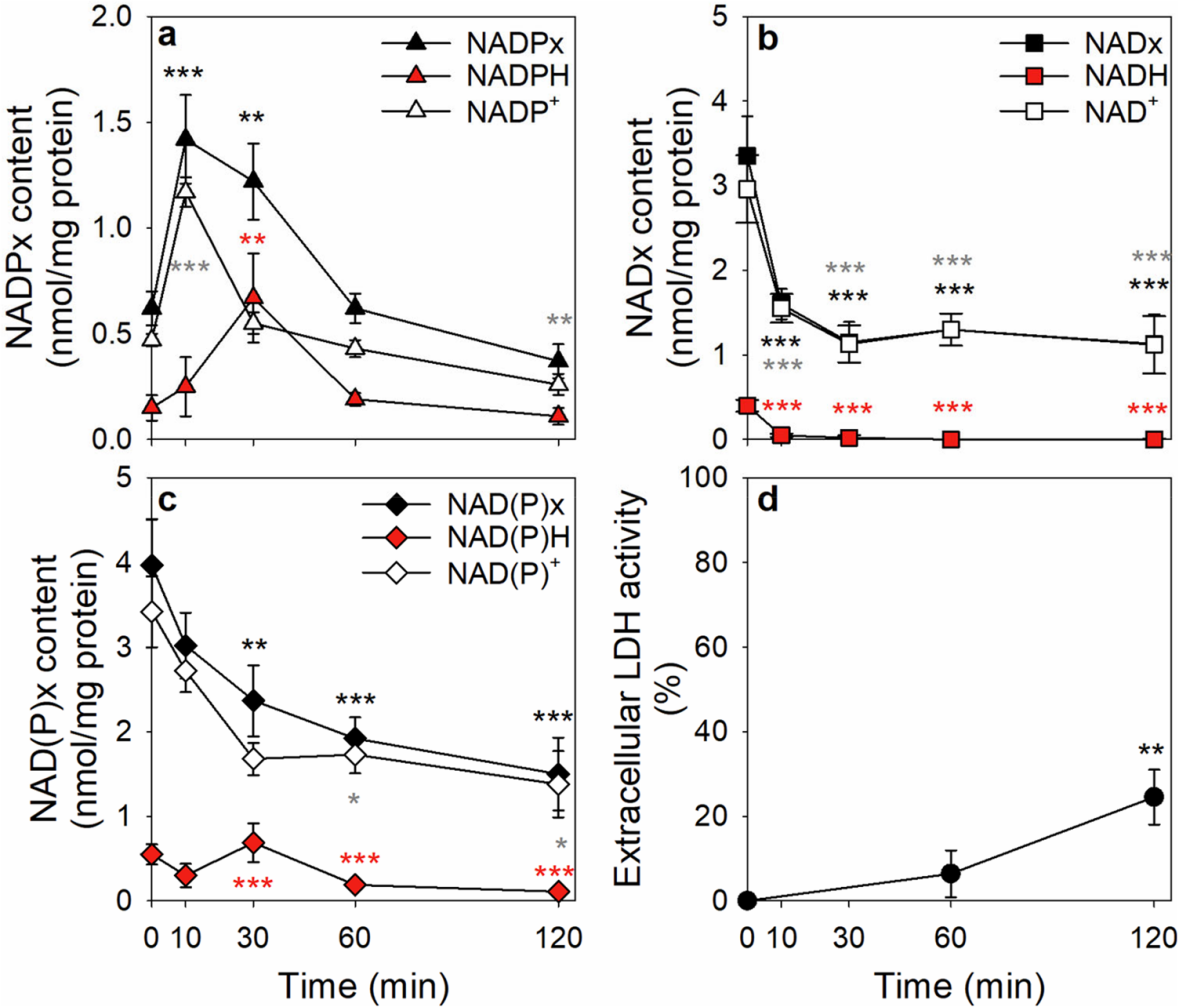
Consequences on the nicotinamide co-substrate levels of cultured astrocytes by an extended incubation after application of H_2_O_2_ and G6PDi-1. The cells were incubated with 100 µM H_2_O_2_ plus 30 µM G6PDi-1 for up to 120 min before the cellular contents of NADPx (**a**) and NADx (**b**) as well as the respective reduced and oxidized redox partners were determined. In addition, the contents of the NAD(P)x, NAD(P)H and NAD(P)^+^ were calculated (**c**) from the data shown in panels a and b, and the extracellular LDH activity (**d**; given as percent of the initial cellular LDH activity) was quantified after 60 and 120 min of incubation. The initial specific NADPx content amounted to 0.62 ± 0.06 nmol/mg protein (with NADPH accounting for 24% of the NADPx pool) and the initial specific NADx content to 3.35 ± 0.47 nmol/mg protein (with NADH accounting for 12% of the NADx pool). The initial protein content of the cultures was 116 ± 6 µg/well and the initial cellular LDH activity was 131 ± 11 nmol/(min x well). The data shown represent the means ± SD of values determined in three independent experiments performed on independently prepared cultures. The significance of differences (ANOVA) compared to the data obtained for the initial time point (0 min) is indicated by *p < 0.05, **p < 0.01 and ***p < 0.001 in the colour of the symbols used to indicate the specific contents of the quantified substances

To test whether the oxidative stress-induced increase in the cellular NADPx content and the accompanied decrease in the cellular NADx content were maintained during incubations for longer than 30 min, the cells were exposed to H_2_O_2_ plus G6PDi-1 for up to 120 min. The results obtained for the initial 30 min of incubation (Fig. 4) were almost identical to those presented above (Fig. 3f, i, l). However, during longer incubations the elevated specific NADPx content of stressed astrocytes was not maintained, but it declined during an extended incubation of 60 min and 120 min to values similar to and even lower than the initial cellular NADPx content, respectively (Fig. 4a). The reestablished high NADPH content found 30 min after the onset of the incubation was also not maintained and declined concomitantly with the overall loss in cellular NADPx during the longer incubation (Fig. 4a). In contrast to NADPx, the lowered specific NADx content found after 30 min incubation remained at the lowered level during the entire incubation period of up to 120 min (Fig. 4b). Accordingly, the specific NAD(P)x content was lowered by around 60% during the 120 min incubation (Fig. 4c), due to the loss in cellular NADPx (Fig. 4a).

Measuring the extracellular LDH activity as an indicator for a potential compromised cell viability revealed that the cell membrane had not been damaged after 60 min of incubation. However, after 120 min a significant increase of the extracellular LDH activity to around 25% of the initial cellular activity was found (Fig. 4d), indicating that the incubation conditions used caused a delayed cell toxicity.

### Test for NAD Kinase Activity in Cultured Astrocytes

**Fig. 5:**
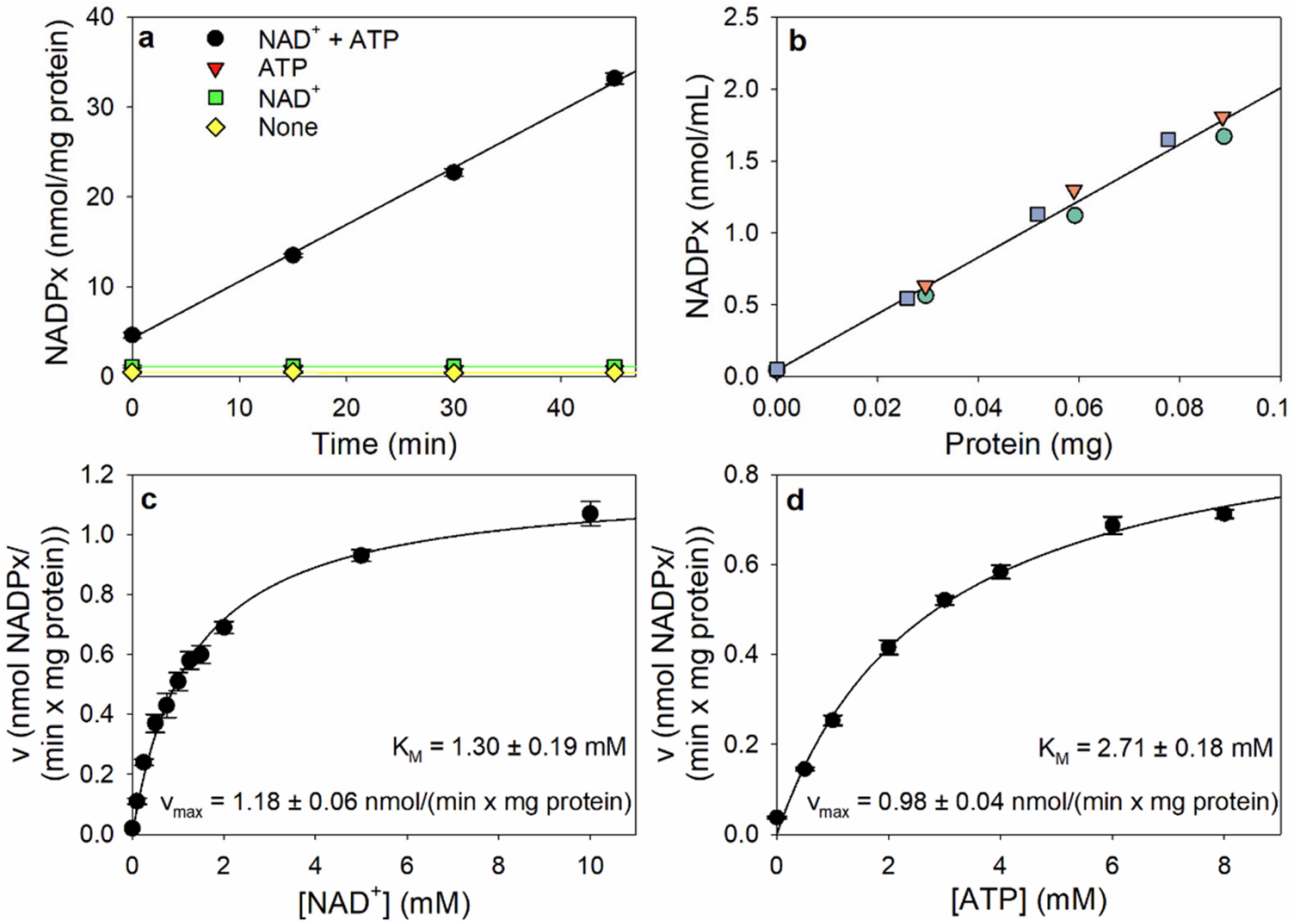
Enzymatic properties of the NADK in lysates of cultured astrocytes. **a** Time-dependency: Lysates were incubated for up to 45 min without or with the substrates NAD^+^ and/or ATP. The mean protein content of the lysates amounted to 56 ± 3 µg/well. **b** Lysate volume-dependency: Volumes of lysate ranging to up to 300 µL were incubated with both substrates for 30 min. The relationship between the activity measured and the protein content of the respective lysate is shown for lysates from three experiments (represented by different colours). The mean protein content of the lysates amounted to 57 ± 4 µg/well. **c** Substrate-dependency for NAD^+^: Lysate samples were incubated with the indicated concentrations of NAD^+^ in the presence of 6 mM ATP for 30 min. The mean protein content of the lysates amounted to 53 ± 2 µg/well. **d** Substrate-dependency for ATP: Lysate samples were incubated with the indicated concentrations of ATP in the presence of 2 mM NAD^+^ for 30 min. The mean protein content of the lysates amounted to 53 ± 2 µg/well. All data shown represents means *±* SD of values determined in three independent experiments performed on the lysates of independently prepared cultures. The K_M_ values represent the calculated concentrations of the substrates that give half-maximal enzymatic velocity of NADK for the lysates investigated

NADP^+^ is known to be generated by NADK-mediated phosphorylation of NAD^+^ [32, 33]. To test for the presence of the enzyme in cultured astrocytes, the supernatant of a hypotonic lysate of the cultures was incubated with the substrates NAD^+^ and ATP and the generation of NADPx was determined. The detectable NADPx content increased proportional to time in the presence of NAD^+^ plus ATP, while no NADPx accumulation was observed for incubations without one or both substrates (Fig. 5a). Furthermore, the NADPx accumulation was proportional to the amount of lysate applied (Fig. 5b). Analysis of the substrate concentration-dependencies revealed that the NADK in lysates from astrocyte cultures has a maximal specific activity of around 1 nmol/(min x mg protein) (Fig. 5c, d). The calculated concentrations of NAD^+^ and ATP that give half-maximal enzymatic velocity of NADK (K_M_ values) for the lysates investigated are 1.3 mM and 2.7 mM, respectively (Fig. 5c, d).

### Effects of a Thionicotinamide-Treatment on the Oxidative Stress-Induced Increase in the NADPx Content in Cultured Astrocytes

**Fig. 6:**
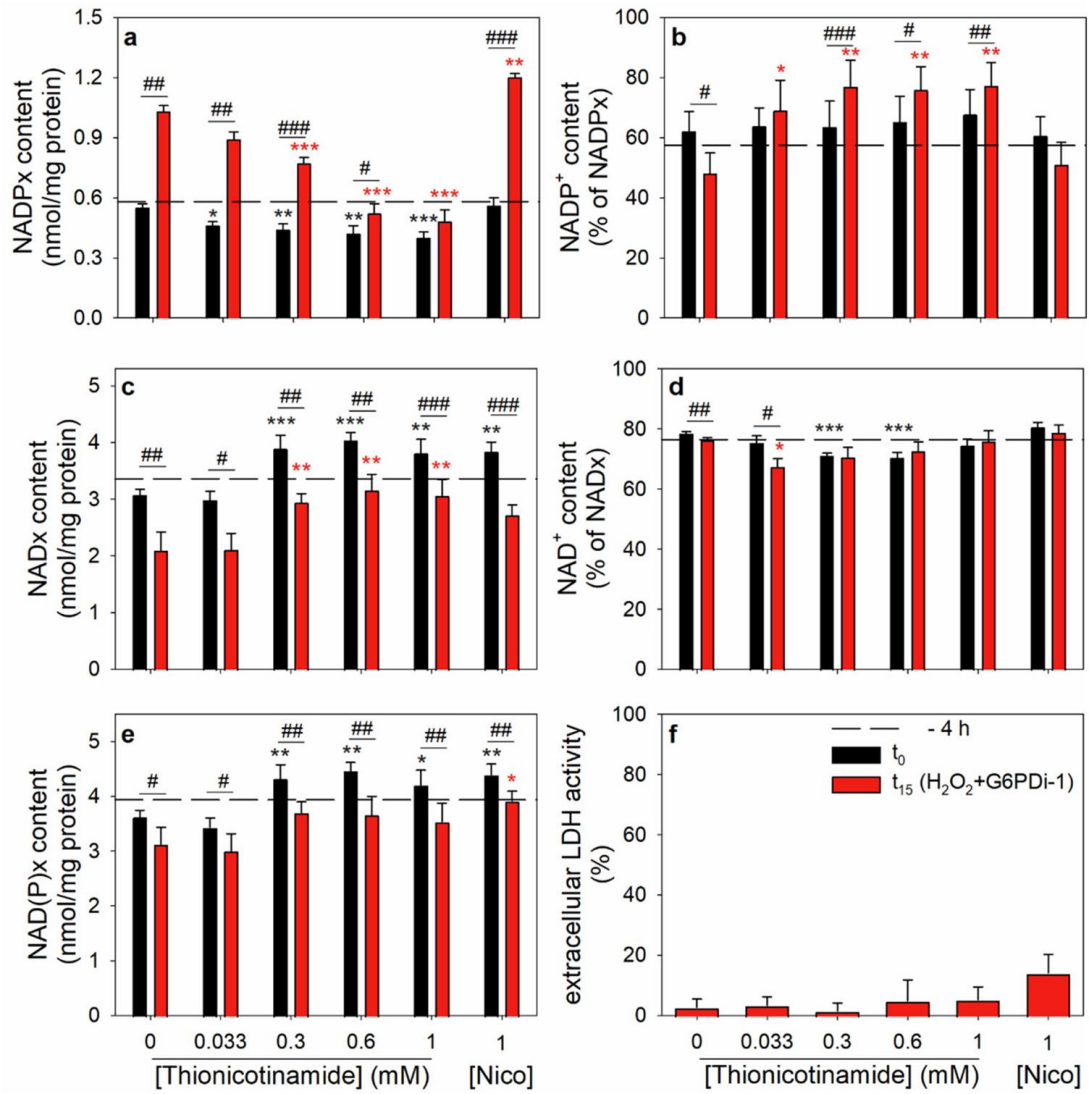
Effects of a preincubation with thionicotinamide or nicotinamide on the NAD^+^ phosphorylation in astrocytes during oxidative stress. The cells were preincubated for 4 h in culture medium without or with the given concentrations of thionicotinamide or with 1 mM nicotinamide (Nico). Subsequently, the cells were incubated with 100 µM H_2_O_2_ and 30 µM G6PDi-1 for 15 min in the presence of the concentrations of thionicotinamide or nicotinamide that had been applied for the respective preincubations. The specific cellular contents of NADPx (**a**) and NADx (**c**) were determined before (t_0_, black bars) and after (t_15_, red bars) the 15 min main incubation and used to calculate the specific NAD(P)x values (**e**). The percental contributions of the oxidized compounds of the respective redox pairs were determined for NADPx (**b**) and NADx (**d**). In addition, the extracellular LDH activity (**f**, given as percent of the initial cellular LDH activity) was quantified after the 15 min main incubation. The values for the initial contents before the preincubation (−4 h) are indicated by the black dashed lines in panels a-e. The initial specific NADPx content amounted to 0.58 ± 0.02 nmol/mg protein (with NADPH accounting for 43% of the NADPx pool) and the initial specific NADx content to 3.36 ± 0.16 nmol/mg protein (with NADH accounting for 24% of the NADx pool). The initial protein content of the cultures was 120 ± 18 µg/well and the initial cellular LDH activity was 132 ± 39 nmol/(min x well). The data shown represent the means ± SD of values determined in three independent experiments performed on independently prepared cultures. The significance of differences (ANOVA) compared to the data obtained for the condition with 0 mM thionicotinamide is indicated by *p < 0.05, **p < 0.01 and ***p < 0.001 in the colour of the respective condition. The significance of differences (t-test) comparing the contents determined before (black) and after (red) the 15 min main incubation with H_2_O_2_ plus G6PDi-1 is indicated by ^#^*p* < 0.05, ^##^*p* < 0.01 and ^###^*p* < 0.001

To investigate the involvement of NADK in the observed elevation of the cellular NADPx content during oxidative stress, cultured astrocytes were preincubated with thionicotinamide for 4 h to enable them to take up this precursor and use it for the synthesis of the NADK inhibitor thio-NADP [44, 45]. After the preincubation, the cells were washed and incubated with 100 µM H_2_O_2_ and 30 µM G6PDi-1 for 15 min in the presence of thionicotinamide or nicotinamide in the concentrations used for the respective preincubations before the contents of cellular NADx and NADPx were determined. A preincubation for 4 h without thionicotinamide or with nicotinamide did not affect the cellular NADPx content (Fig. 6a) compared to the initial value (indicated by the dashed line), while a preincubation with thionicotinamide lowered the cellular NADPx content slightly but significantly in a concentration-dependent manner by up to 27% as determined for a preincubation with 1 mM thionicotinamide (Fig. 6a, black bars). The contribution of NADP^+^ to the overall NADPx content was not altered for any of the applied preincubation conditions (Fig. 6b, black bars) compared to the initial value (Fig. 6b, dashed line).

During the preincubation in the presence of 0.3, 0.6 and 1 mM thionicotinamide or 1 mM nicotinamide, the specific cellular NADx content (Fig. 6c, black bars) and the calculated NAD(P)x content (Fig. 6e, black bars) were slightly but significantly increased by around 30% and 20%, respectively, compared to the initial values (dashed lines). The percental contribution of NAD^+^ to the NADx redox pair was around 80% and was hardly affected by the different preincubation conditions (Fig. 6d, compare black bars with dashed line). The increase in the detectable NADx level during preincubation with nicotinamide is likely to be caused by improved synthesis of NAD^+^ from this precursor as previously reported [46]. For thionicotinamide-treated cells, the thio-NAD formed from thionicotinamide [44, 45] may partially contribute to the increased levels of NADx determined as thio-NAD reacts in the NADx cycling assay used with low velocity (data not shown).

Application of H_2_O_2_ and G6PDi-1 for 15 min to the preincubated cells caused a doubling in the cellular NADPx content (from 0.58 to more than 1 nmol/mg protein) in astrocytes which had been preincubated without thionicotinamide or with 1 mM nicotinamide (Fig. 6a, red bars). This oxidative stress-induced increase in the cellular NADPx content was lowered in a concentration-dependent manner by a preincubation with thionicotinamide. 1 mM thionicotinamide completely prevented the oxidative stress-induced increase in the cellular NADP^+^ content (Fig. 6a). In addition, the presence of thionicotinamide, but not of nicotinamide, increased the percental amount of NADP^+^ contributing to the NADPx redox pair (from initially 57% to up to 77%) (Fig. 6b, red bars).

During the main incubation, the cellular NADx content (Fig. 6c) and the calculated NAD(P)x content (Fig. 6e) were significantly lowered by around 20–30% and 15%, respectively. However, for incubations with at least 0.3 mM thionicotinamide the NADx content remained significantly higher than the NADx content of astrocytes that had been incubated in the absence of thionicotinamide (Fig. 6c). The contribution of NAD^+^ to the NADx content was hardly affected by the application of H_2_O_2_ and G6PDi-1 for 15 min (Fig. 6d). The viability of the cells was not compromised as demonstrated by the low extracellular activity of the cytosolic enzyme LDH (Fig. 6f).

## Discussion

To study the interplay between the main redox pairs in cultured astrocytes, we have quantified the reduced and oxidized components of the redox pairs GSx (GSH plus GSSG), NADPx (NADP^+^ plus NADPH) and NADx (NAD^+^ plus NADH) for different incubation conditions. In untreated astrocyte cultures, the average specific contents of the cellular redox pairs that had been determined in a large number of independent experiments and cultures were in a similar range to values previously reported by us and other groups for the contents of GSx [47–50], NADPx [18, 38, 51, 52] and NADx [18, 38, 46, 51]. Differences between the reported total contents of the three redox pairs in cultured astrocytes are likely to be caused by different culturing conditions as the composition of culture media has been reported to have substantial effects on the astrocytic contents of at least GSx [47, 53] and NADx [38, 46]. Some heterogeneity in the specific contents of cellular components of the investigated redox pairs was also found between individual astrocyte cultures that were prepared and maintained under identical conditions (Fig. 1). This heterogeneity appears to be an intrinsic problem of such cultures as similar heterogeneities were also reported for the specific contents of ATP and creatine phosphate [54], for the consumption of glucose and for the release of lactate and pyruvate [14] from astrocyte cultures.

On average, the reduced partners of the three redox pairs, GSH, NADPH and NADH, accounted for 97 ± 3%, 37 ± 14% and 28 ± 10% of the total contents, clearly demonstrating a substantial difference in the ratio of the reduced to the oxidized partners of these redox pairs. The very high ratio of GSH to GSSG in untreated cultured astrocytes is consistent with literature data [18, 27, 50, 55] and reflects the highly efficient NADPH-dependent reduction of GSSG by glutathione reductase in unstressed astrocytes [7]. The predominance of NAD^+^ in the NADx redox pair confirms literature data for cultured astrocytes [18, 38, 46, 51, 56] and reflects the availability of NAD^+^ to efficiently serve as electron acceptor for oxidoreductases of catabolic pathways, such as the glycolytic glyceraldehyde-3-phosphate dehydrogenase [8].

In the total number of 28 measurements analysed for our study, NADPH accounted on average for around 40% of the NADPx redox pair in untreated astrocytes with values determined for individual cultures ranging from 13% to 66% NADPH. The average percental content of NADPH is lower than those previous reported for cultured astrocytes which found NADPH to represent 70% [18], 60% [52], 55% [51] and 44% [38] of the NADPx content. Nevertheless, the NADPx redox pair is more strongly reduced than the NADx redox pair, consistent with literature data [8, 18]. This appears to be sufficient to enable the cultures to use NADPH as electron donor for the regeneration of GSSG via glutathione reductase and as substrate for reductive biosyntheses [7, 8, 17].

Several metabolic parameters of cultured astrocytes have recently been analysed for potential alteration with cultures age. For example, with an increasing astrocyte culture age a decline in the specific cellular content of creatine phosphate [54] and in the pyruvate release [14] was observed, while the specific cellular contents of ATP, ADP and AMP [54, 57], the specific glucose consumption and lactate release [14] as well as the cell-dependent WST1 reduction capacity [20] were not altered with increasing culture age. Analysis of the determined specific contents of the three redox pairs for a potential alteration with the cultures age revealed that the specific contents of NADx and NADPx were not altered with culture age, while the specific GSx content declined by around 30% between cultures of an age of 14 d and 28 d. The percental contribution of the oxidized and reduced partners of GSx and NADPx was not significantly modified by the culture age, while the ratio of the contents of NAD^+^ and NADH was altered to a more reduced state with increasing culture age. This may reflect an impaired consumption of NADH-derived electrons in aged cultures, but this would contrast the reported shift towards a more oxidized ratio of the NADx redox pair in rodent brain with ageing [46, 58].

To investigate the interplay of the three main redox pairs in cultured astrocytes during oxidative stress, we applied H_2_O_2_ either alone or in combination with the specific PPP inhibitor G6PDi-1 [19, 43]. Extracellular H_2_O_2_ was removed by the cells with a half-time in the low minute range as previously reported [27, 41]. The involvement of the GSx redox system in the peroxide clearance is demonstrated by the transient glutathione peroxidase-dependent increase in cellular GSSG to around 25% of the GSx content, consistent with literature data [27, 59]. The presence of the PPP inhibitor G6PDi-1 significantly slowed the peroxide clearance as demonstrated by a 30% prolonged half-time, consistent with data previously obtained for glucose-free incubations of astrocytes with H_2_O_2_ [27]. The inhibition of the PPP by G6PDi-1 [19] will prevent PPP-dependent regeneration of NADPH which is required for the reduction of GSSG to GSH by glutathione reductase [7] and will thereby severely slow GSH dependent peroxide reduction by glutathione peroxidase [27, 60]. As expected, the peroxide clearance in astrocytes was only moderately slowed by impairment of the GSH- and NADPH-dependent detoxification system as catalase-dependent H_2_O_2_ detoxification is able to compensate at least in part for the loss of the GSH-dependent peroxide clearance [27, 59]. However, the co-application of G6PDi-1 with H_2_O_2_ caused an almost complete oxidation of cellular GSH to GSSG within 5 min of incubation and the high initial GSH to GSSG ratio was only re-established after 30 min of incubation, confirming that G6PDi-1 is a powerful inhibitor of PPP-dependent NADPH regeneration [19] and that the PPP is essential for rapid GSSG reduction during peroxide detoxification by astrocytes [27, 61].

The strong accumulation of GSSG after a coapplication of H_2_O_2_ with G6PDi-1 to astrocytes was accompanied by severe alterations in the specific contents of NADPx and NADx as demonstrated by the strong increase in the cellular NADPx content on the expense of the NADx content. This suggests that the oxidative stress condition applied induced the phosphorylation of NAD^+^ to NADP^+^ in astrocytes. The enzyme responsible for the phosphorylation of NAD^+^ to NADP^+^ in mammalian cells is NADK [32, 33]. NAD^+^- and ATP-dependent NADPx formation was confirmed for lysates of cultured astrocytes. The K_M_ values determined for the two substrates, NAD^+^ (1.30 ± 0.19 mM) and ATP (2.71 ± 0.18 mM), are quite similar to the values (NAD^+^: 0.5 mM, 0.54 mM; ATP: 3.3 mM, 4 mM) reported for rat brain NADK [35] and human NADK [62]. These K_M_ values are also in the range of the cytosolic concentrations of the two substrates in cultured astrocytes (1.5 mM ATP [63]; 0.73 mM NAD^+^ [46]), supporting the view that astrocytic NADK is responsible for the phosphorylation of NAD^+^ to NADP^+^ under the conditions studied.

Thionicotinamide was applied to cultured astrocytes to confirm that NADK is responsible for the accumulation of NADPx during oxidative stress. This precursor of a NADK inhibitor is taken up into cells, converted to thio-NAD and subsequently phosphorylated to thio-NADP by NADK which serves as an efficient inhibitor of NADK [44, 45, 64]. The application of thionicotinamide caused a concentration-dependent inhibition of the NADPx accumulation in astrocytes that had been treated with H_2_O_2_ plus G6PDi-1, demonstrating that indeed NADK is responsible for the oxidative stress-induced phosphorylation of NAD^+^ to NADP^+^. Concerning the specificity of the inhibitory profile of a thionicotinamide-treatment it should be mentioned that thio-NADP has been reported to also inhibit human G6PDH in addition to NADK [44]. However, an inhibition of G6PDH after application of thionicotinamide to cultured rat astrocytes is unlikely to take place under the conditions used as the ratio of NADP^+^ to NADPH was not affected by a 4 h thionicotinamide preincubation.

Currently, the mechanism which is involved in the activation of astrocytic NADK during oxidative stress remains unclear. The shift in the cellular ratio of NADP^+^ to NADPH by oxidation of NADPH to NADP^+^ during GSSG reduction may activate NADK, consistent with literature data showing inhibition of NADK by a high NADPH concentration and activation by lowering the NADPH concentration [65]. The formation of additional NADP^+^ on the expense of NAD^+^ by activated NADK would increase the cellular concentration of available NADP^+^, thereby potentially accelerating the reactions of NADPH regenerating enzymes and improving NADPH- and GSH-dependent detoxification processes. Accordingly, a decreased NADK activity has been shown to increase the sensitivity of cells against oxidative stress [66].

Although the applied extracellular H_2_O_2_ had been completely removed by the cells within 15 min, substantial amounts of GSSG were still detectable in the G6PDi-1-treated astrocytes, demonstrating that GSH regeneration was still impaired, clearly confirming the importance of a functional oxidative part of the PPP for efficient astrocytic NADPH regeneration during peroxide detoxification. As soon as the applied peroxide had been cleared by the cells within 15 min, no further increase in cellular NADPx was observed. However, the contribution of NADPH to the NADPx pair increased only slowly and it took another 15 min before the initial ratio of NADPH to NADP^+^ was found. Similarly, 30 min after application of H_2_O_2_ the initial high ratio of GSH to GSSG had also been reestablished. This suggests that under the conditions used PPP-independent NADP^+^ reducing enzymes provided electrons in form of NADPH - although less efficiently than the dehydrogenases of the PPP - that were used for GSSG reduction by glutathione reductase. Likely candidates for such enzymes are the cytosolic NADP^+^-dependent isocitrate dehydrogenase as well as the malic enzyme which are both expressed in cultured rat astrocytes [21, 22].

During severe oxidative stress, the cellular NADPx content was strongly increased on the expense of the NADx pool in astrocytes. The increase in the cellular NADPx pool was transient and during incubations longer than 30 min the NADPx pool was lowered to values similar to those of untreated cells. In contrast, the lowered NADx values remained stable on the lower level during extended incubation. This suggests that the delayed decline in cellular NADPx was not caused by hydrolysis of NADPx to NADx, but that rather another NADPx consuming enzyme is responsible for the removal of the elevated NADPx content in astrocytes that had been treated with H_2_O_2_ plus G6PDi-1. This enzyme could be the glycohydrolase CD38 [67, 68] which has been strongly connected with the regulation of the NAD^+^ level in astrocytes during inflammation [69, 70], but has actually a lower K_M_ value for NADPx than for NADx [71]. CD38, which liberates nicotinamide from NADP^+^, may also be responsible for the observed lowering of the cellular NADPx content during a preincubation of the cells with thionicotinamide as previously also reported for colon cancer cells [44]. However, in preliminary experiments the application of the CD38 inhibitor compound 78c [72] did not substantially slow down the removal of the initially elevated NADPx content after oxidative stress (data not shown), suggesting that other enzymes are involved in the observed removal of NADPx in cultured astrocytes under the conditions studied. The lowered NADPx content found after preincubation with thionicotinamide may be the consequence of a disturbance of the normal turnover of NADPx by an impairment of NADK-mediated generation of NADPx, while the cellular consumption of NADPx is not altered.

As most of the cellular GSH is localized in the cytosol [73], the transient accumulation of GSSG after induction of oxidative stress by application of H_2_O_2_ is likely to mainly be the consequence of peroxide reduction by cytosolic glutathione peroxidase. This is supported by the strong acceleration of cellular GSSG accumulation by coapplication of the PPP inhibitor G6PDi-1. Mitochondria of astrocytes have been reported to contain GSx [73] and NADx [74] and also NADPx needs to be present in astrocytic mitochondria as substrate for enzymes such as the mitochondrial NADP^+^-dependent isocitrate dehydrogenase [21, 22]. However, a substantial contribution of the mitochondrial redox pairs in the observed alterations in the total cellular redox pairs during the applied stress conditions appears to be rather unlikely. Accordingly, of the two known isoforms of NADK, cytosolic and mitochondrial NADK [32, 33], the cytosolic NADK appears to be mainly responsible for the observed increase in cellular NADPx. Nevertheless, additional research is now necessary to investigate the potential effects of oxidative stress on the mitochondrial pools of GSx, NADPx and NADx and on a potential phosphorylation of NAD^+^ to NADP^+^ in astrocytic mitochondria. Further studies are also required to elucidate whether an increased cellular NADPx level during oxidative stress is beneficial for astrocytes, for example by accelerating NADPH-dependent GSH redox cycling, and/or whether a lowered NADx level may impair the oxidation of energy substrates.

In conclusion, the data presented demonstrate a strong interaction of the three main redox pairs during the defence of astrocytes against oxidative stress. In addition to the NADPH- and PPP-dependent regeneration of GSH from the GSSG that is accumulated during peroxide detoxification, the available cellular NADPx pool is increased by NADK-mediated phosphorylation on the expense of the NADx pool.

## Data Availability

Enquieries on original data should be directed to the corresponding author.
